# Comparative study of halo-vest reduction and skull traction reduction in the treatment of cervical fracture dislocation in patients with ankylosing spondylitis

**DOI:** 10.3389/fsurg.2023.1129809

**Published:** 2023-05-09

**Authors:** Liang Wang, Haibin Wang, Can Wang, Bangke Zhang, Haisong Yang, Xuhua Lu

**Affiliations:** ^1^ School of Health Science and Engineering, University of Shanghai for Science and Technology, Shanghai, China; ^2^ Department of Orthopaedics, Second Affiliated Hospital of Naval Medical University, Shanghai, China; ^3^ Department of Orthopaedics, Affiliated Hospital of North Sichuan Medical College, Sichuan, China

**Keywords:** ankylosing spondylitis (AS), halo-vest immobilization, skull traction, cervical spine trauma, American Spinal Injury Association (ASIA) impairment scale, cervical traction

## Abstract

**Background:**

This study aimed to investigate the safety and efficacy of the halo-vest in the treatment of cervical fracture in patients with ankylosing spondylitis (AS) and kyphosis.

**Methods:**

From May 2017 to May 2021, 36 patients with cervical fractures with AS and thoracic kyphosis were included in this study. The patients with cervical spine fractures with AS underwent preoperative reduction by halo-vest or skull tractions. Instrumentation internal fixation and fusion surgery were then performed. The level of cervical fractures, the operative duration, blood loss, and treatment outcomes were investigated preoperatively and postoperatively.

**Results:**

A total of 25 cases were included in the halo-vest group and 11 cases were included in the skull tractions group. The intraoperative blood loss and the surgery duration were significantly less in the halo-vest group than in the skull traction group. A comparison of American Spinal Injury Association scores at admission and final follow-up showed that the neurological function of patients improved in both groups. All patients had reached solid bony fusion during the follow-up.

**Conclusion:**

This study presented a unique approach to use halo-vest treatment fixation of unstable cervical fracture in patients with AS. The patient should also have early surgical stabilization with a halo-vest to correct spinal deformity and avoid worsening of neurological status.

## Introduction

Ankylosing spondylitis (AS) is a chronic autoimmune disease involving axial joints, peripheral joints, and even extra-articular organs. Compared with the general population, patients with AS are about four times more likely to experience cervical spine fractures (1, 2). Due to the ossification of paravertebral ligaments and intervertebral discs, osteopenia, osteoporosis, and joint erosion, the patient's activities are limited, the spine may be rigid and deformed, and the stability function is poor (3). Therefore, the risk of spinal fracture is high, especially in the C5–7 segment of the cervical spine, which is prone to low-energy fractures (4, 5). Even a low-energy accident can result in a serious neurological injury (6). Most neurological injuries accompany mechanical injury or prior to stabilization (halo-vest or skull traction), and the long lever arm is extremely unstable compared to cervical spine fractures in healthy individuals (7–9). Neurological deficits may accompany vertebral fractures or displacements (especially in hyperextension injuries) (10). In addition, AS patients with highly unstable mechanical injuries are at higher risk for secondary neurological injury. Morbidity and mortality are higher in older AS patients than younger AS patients with similar or more severe injuries (11).

When vertebral fractures occur in AS patients, the spine becomes very unstable, and primary and secondary nerve damage and progressive deformity are avoided (12). Skull traction is a widely used for reduction and immobilization in patients with cervical fracture dislocation (13). However, skull traction therapy is challenging because patients with AS may have kyphosis, primarily when the kyphosis is located in the thoracic spine (14). Due to unstable fractures and dislocation of cervical spine in AS, skull traction has the risk of aggravating neurological symptoms (15). A halo-vest is an effective adjuvant tool for correcting spinal deformities (16, 17). After reducing supine or prone fractures, the halo-vest is used to externally fix the patient, providing the most potent external fixation for the upper cervical spine.

However, the optimal preoperative traction approach for cervical spine fractures in AS patients remains controversial. This study reports on our experience in treating patients with AS cervical spine fractures to stabilize the fracture to prevent neurological deterioration and partially correct the deformity from restoring the preinjury state.

## Material and methods

**Figure 1:**
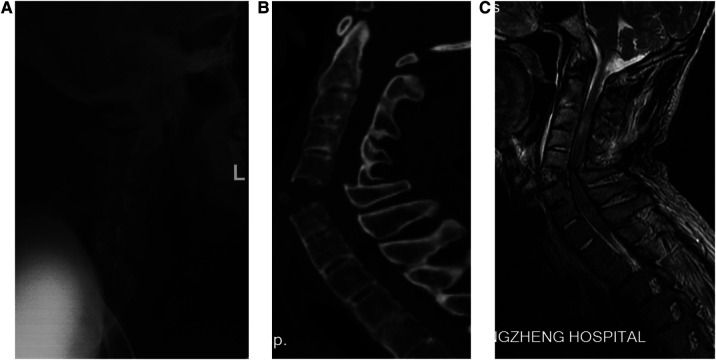
A 57-year-old man with AS and thoracic kyphosis and was diagnosed as fracture dislocation at C5/6. (**A**) Lateral radiography reveal a severe dislocated fracture at C5–6 levels with significant widen at C5–6 intervertebral space and so-called bamboo spine resulting from ankylosing spondylitis. (**B**) Sagittal CT showing fracture dislocation at C5/6. (**C**) MRI demonstrating increased signal segmentally at the level of the fracture indicate total segmental disruption. AS, ankylosing spondylitis; MRI, magnetic resonance imaging.

From May 2017 to May 2021, 36 AS and kyphosis patients with cervical spine fractures received halo-vest or skull traction to assist closed preoperative reduction. This study utilized a retrospective chart review and radiology follow-up study, including plain radiographs and magnetic resonance imaging (MRI) (Figures 1A–C). The fractures of all AS patients involved the anterior column to the posterior column, along with the fractures of ossified anterior and posterior ligamentous complexes and the surrounding tissue. All 36 patients were diagnosed with AS preoperatively, and 24 received AS medication. The reduction was assessed by x-ray examination of lateral cervical vertebrae beside bed. Anatomical reduction was defined as a <1 mm distance between two surgically restored vertebral bodies; reduction success was defined as a 1- to 3-mm distance, and reduction failure was defined as a >3 mm distance (18).

### Halo-vest procedure

**Figure 2:**
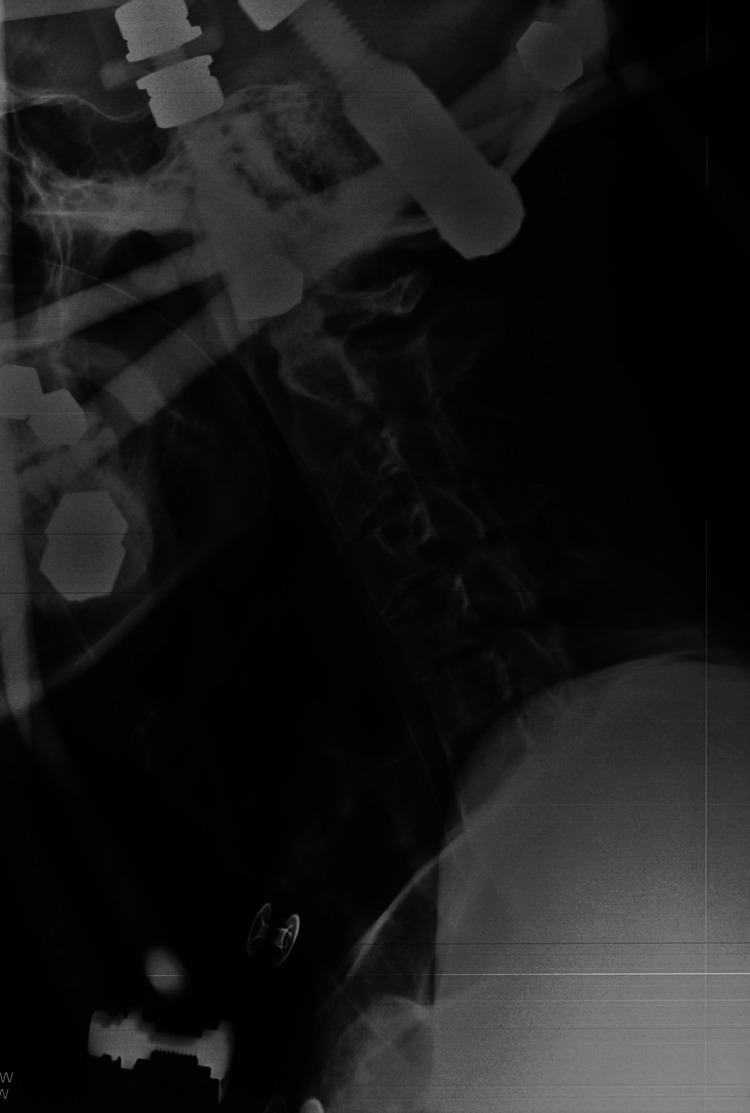
Reduction of cervical fractures in patients with AS from the perspective of a C-arm radiograph system. AS, ankylosing spondylitis.

Halo-vest reduced the fracture, and the patient underwent x-ray examination after reduction. Halo-vest devices are applied in a standardized manner based on usual clinical methods. First, the patient's head is supported by a cervical collar and placed in a supine position. Next, the surgeon lifted the behind of the head ring upward, and simultaneously the assistant moved down the front of the head ring. The steps were repeated until the patient restored the preinjury status. The surgeon and assistant then tightened each pin using a torque screwdriver. Finally, plain lateral x-rays were taken to evaluate the cervical spine under conditions of halo-vest fixation (Figure 2). Halo-vest immobilization lasted for a mean period of 2 ± 1 days before operative stabilization.

**Figure 3:**
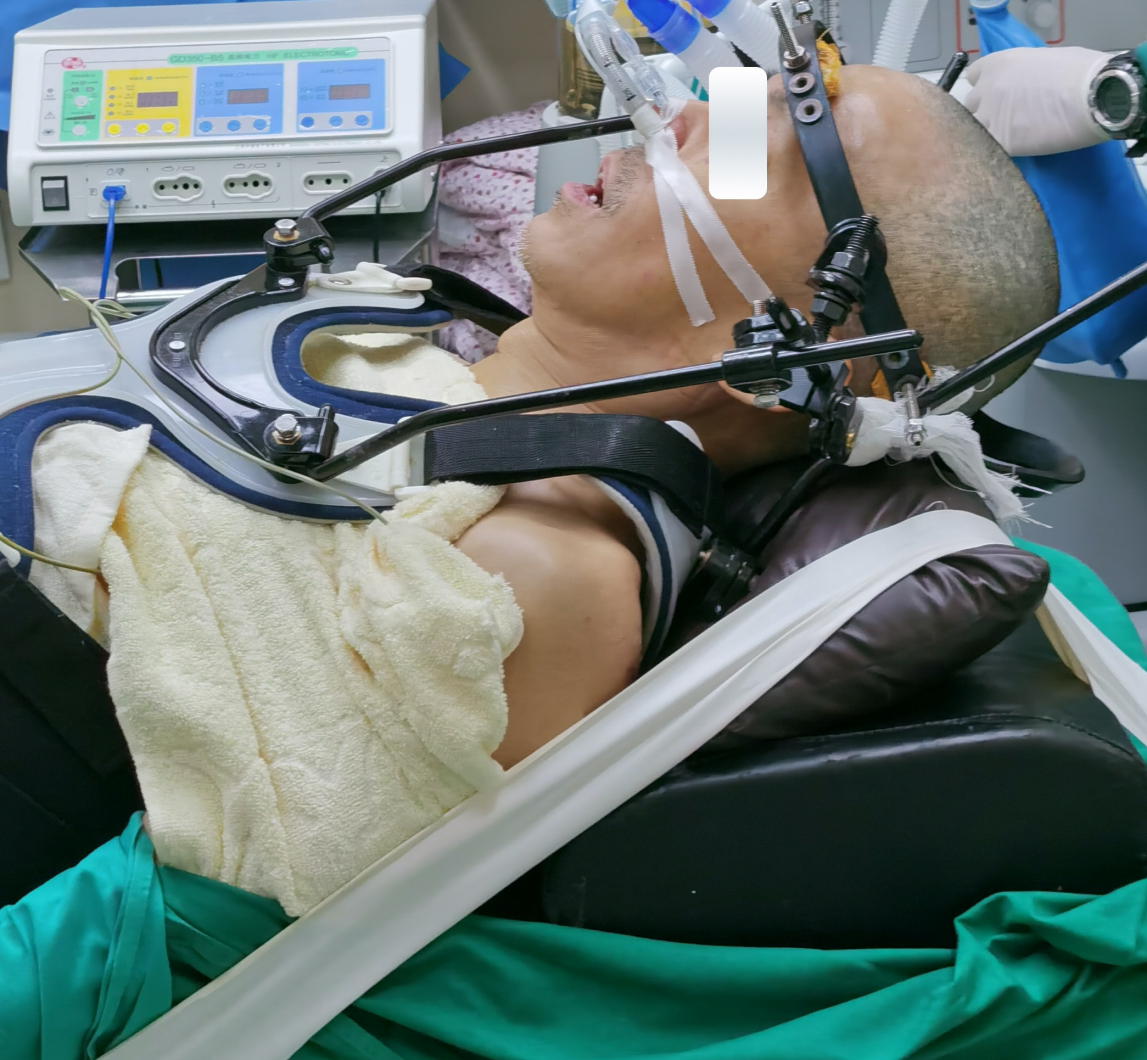
Nasotracheal intubation and sitting position with halo-vest.

Surgical treatment slows or stabilizes a patient's neurological deficit and instability. With the patient kept awake, nasotracheal intubation was used, followed by general anesthesia, and the patient was carefully placed in a sitting position using a halo-vest (Figure 3). To avoid displacement of the patient's fracture site, the surgeon needs to hold the halo-vest and transfer the patient from the stretcher to the operating table. If preoperative reduction failed, it was repeated after repositioning via regulation of the length of the anterior and posterior bars of the halo-vest under neurophysiologic monitoring until satisfactory restoration was achieved.

### Skull tractions

Skull tractions with a traction weight of 4–6 kg were performed on patients with fracture dislocations after admission. The traction direction was in line with the neck to reduce dislocation or fracture dislocation. Immobilization in flexion by placing sandbags under occiput reduces the fracture and prevents cord damage. The patients received x-ray examination of lateral cervical vertebrae beside bed before nasoendotracheal intubation and induction of general anesthesia. The skull tractions lasted for a mean period of 2 ± 1 days before operative stabilization.

### Operative approach

**Figure 4:**
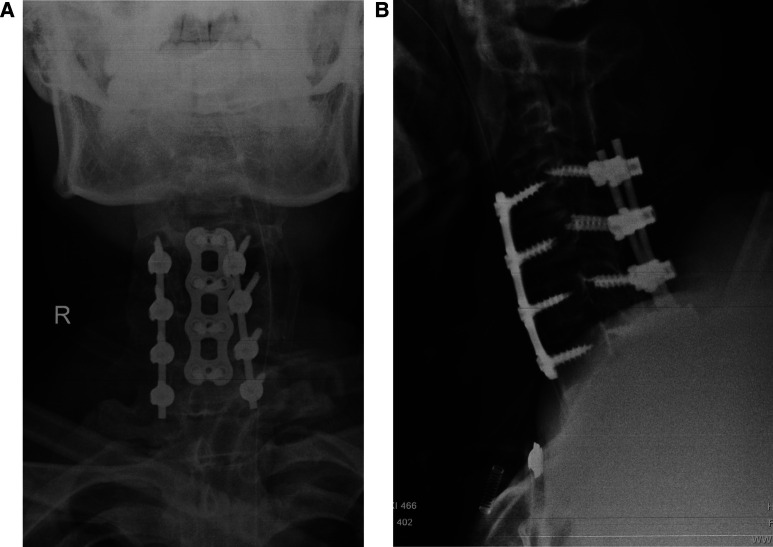
Postoperative plain radiography (**A,B**).

The posterior approach as the first choice was operated to restore the spinous process and performed with at least two segments fixed both upper and lower. If the fracture dislocations were reduced, the patients were treated with the simple posterior instrumentation internal fixation and fusion surgery. For those patients with excessive separation in anterior fractures or apparent anterior compression, anterior–posterior instrumentation internal fixation and fusion surgery were adopted. Intraoperative neurophysiologic monitoring was performed. Finally, x-ray films were used to check the patient's fracture reduction and fixation maintenance (Figures 4A,B). Patients were treated with corresponding surgeries.

### Follow-up

One day post-operation, the patients were allowed to sit with a Philadelphia collar. The Philadelphia collar was used for 2 months. Patients were monitored for radiographic and neurological outcomes. Complications were also recorded, such as infection, dysphagia, death, screw loosening and breaking, fusion cage subsidence, and rod breakage. The American Spinal Injury Association (ASIA) impairment scale was used to evaluate the neurological status pre-and post-operation. All patients received an x-ray of the whole cervical spine or an MRI of the cervical spine if required.

### Statistical analysis

SPSS 21.0 software (IBM Corp., Armonk, NY, United States) was used for statistical analysis. The *t* test was used for comparison of the mean between the two groups, and the Chi-square test was used for comparison of the ratio between the two groups. *P* <0.05 was considered statistically significant.

## Results

The demographic characteristics of the 36 patients in the two groups are listed in Table 1. From May 2017 to May 2021, 25 cases were in included in the halo-vest group and 11 cases were included in the skull tractions group. A single spinal segment was involved in each patient. The mean age was 58.5 ± 6.1 years, range 49–68 years. Thirty-five AS patients with fracture dislocation of the cervical spine received posterior or combined anterior–posterior approach within 3 days after injury. In the halo-vest group, the fracture levels were odontoid in 2 patients, C2/3 in 3 patients, C4/5 in 5 patients, C5/6 in 13 patients, and C6/7 in 2 patients. Among the skull traction group, 1 patient was diagnosed with odontoid fractures, 2 patients were diagnosed with C2–3 fractures, 1 patient was diagnosed with C3–C4 fractures, 3 patients were diagnosed with C4–C5 fractures, 3 patients were diagnosed with C5–C6 fractures, and 1 patient was diagnosed with C6–C7 fractures (Table 1).

**Table 1:** Baseline characteristics of patients in the two groups.

Characteristics	Halo-vest	Skull traction	*P*
Age (years)	54.56 ± 9.19	48.00 ± 7.14	
Gender			0.913
Male	23 (92.0%)	10 (90.9%)	
Female	2 (8.0%)	1 (9.1%)	
Preoperative ASIA grade			0.220
A	1 (4.0%)	2 (18.2%)	
B	2 (8.0%)	3 (27.3%)	
C	8 (32.0%)	1 (9.10%)	
D	12 (48.0%)	4 (36.4%)	
E	2 (8.0%)	1 (9.10%)	
Postoperative ASIA grade			0.051
A	–	1 (9.1%)	
B	–	1 (9.1%)	
C	1 (4.2%)	2 (18.2%)	
D	15 (62.5%)	2 (18.2%)	
E	8 (33.3%)	5 (45.5%)	
Damaged segment			0.588
OF	2 (8.0%)	1 (9.1%)	
C2/3	3 (12.0%)	2 (18.2%)	
C3/4	−(0.0%)	1 (9.1%)	
C4/5	5 (20.0%)	3 (27.3%)	
C5/6	13 (52.0%)	3 (27.3%)	
C6/7	2 (8.0%)	1 (9.1%)	
Complication	1	2	0.001^*^
Surgery duration (min)	180.36 ± 55.74	238.63 ± 56.78	0.007^*^
Blood loss (ml)	310.40 ± 16.19	415.45 ± 59.05	0.001^*^
Type of reduction			0.356
Successful	2 (8.0%)	1 (9.1%)	
Anatomical	22 (88.0%)	4 (72.7%)	
Failed	1 (4.0%)	6 (18.2%)	
Bone fusions	24	9	0.310
Operative approach			0.861
Combined anterior and posterior approach	16	7	
Posterior approach alone	8	4	

ASIA, American Spinal Injury Association; OF, odontoid fracture.

^*^ *P *< 0.05.

In the present study, satisfactory preoperative reductions occurred in 25 patients in the halo-vest group and in 5 patients in the skull traction group, and no further neurological decline occurred in the follow-up. In the halo-vest group, 16 patients were treated by combined anterior and posterior approach and the others by posterior approach alone. In the skull traction group, 7 patients were treated by a combined anterior and posterior approach and 4 patients by simple posterior approach. The mean surgical duration for patients with halo-vest was 180.36 ± 55.74 min, while it was 238.63 ± 56.78 min for patients with skull traction (*P* < 0.05). The skull traction group had more blood loss (415.45 ± 59.05 ml vs. 310.40 ± 16.19 ml, *P* < 0.05) than the halo-vest group. The present study showed no statistical difference in the operative approach between the halo-vest group and the skull traction group (*P *> 0.05).

All patients were followed up postoperatively: average follow-up period was 12–36 months. Bone fusions were observed in 9 of 11 patients (81%) in the skull traction group and 24 of 25 patients (96%) in the halo-vest group. The differences in ASIA score between groups were not statistically significant before surgery. In the halo-vest group, 1 case was grade A, 2 cases were grade B, 8 cases were grade C, 12 cases were grade D, and 2 cases were grade E. In the skull traction group, two cases were grade A, three cases were grade B, one case was grade C, three cases were grade D, and one case was grade E. Over follow-up, neural function was recovered in all patients. In the halo-vest group, one improved grades from B to C, one improved grades from B to D, eight improved grades from C to D, and six improved grades from D to E. In the skull traction group, one patient with grade A showed no significant neurological recovery, but one with grade A became grade B, one of two grade A became grade B postoperatively, two of three grade B became grade C postoperatively, one of three grade B became grade D postoperatively, one with grade C became grade D postoperatively, and four with grade D became grade E postoperatively (Table 1).

### Complications

Complications occurred in one case with the halo-vest. However, lung pneumonia was diagnosed in one case with halo-vest-assisted closed reduction (ASIA grade A) before surgery. He was unable to undergo surgery and died 9 days after the injury. Surgery-related complications were not observed. In the skull traction group, postoperative complications occurred in two patients (18.2%), including implant failure (*n* = 1) and pneumonia (*n* = 1) (Table 1). The one patient with the AS fracture dislocation experienced early implant failure (screw loosening) 10 days postoperatively due to great local stresses which was related to skull traction treatment that could not completely reduce the AS cervical spine fractures during the operation; this patient required revision surgery and anterior–posterior fixation. Pneumonia in the patient was related to skull traction treatment for a long time in bed.

## Discussion

Compared with the general population, patients with AS are more likely to develop spinal fractures and spinal cord injuries (19). However, the incidence of AS is low, and it is difficult to count such patients; there is a lack of treatment and clinical information for patients with fractures. Therefore, correcting fracture deformities in AS patients is challenging. The closed reduction of the cervical fracture dislocation in patients with AS has been previously described and is now a recognized procedure (20). However, the safety of closed reduction has been the focus of debate for some time. This study compared halo-vest reduction and skull traction for AS cervical spine fractures.

Recently, some studies reported the treatment of spinal fractures in AS patients (21, 22). Longo et al. reported that more than 80% of the patients’ injuries were low-energy cervical spine injuries, which led to the dislocation of the cervical spine structure in AS patients and unstable cervical spine fracture morphology (23). Due to the high risk of fracture and dislocation in AS patients, secondary neurological deterioration and progressive deformity may occur, resulting in a poor clinical prognosis. Therefore, patients with AS cervical spine fractures require close monitoring and early rehabilitation.

The utility of the skull traction is widespread, and they have proven useful for cervical fracture dislocation and temporary stabilization (24). While it can make subsequent operation easier, failure rates are reported to be high. Excessive skull traction could aggravate vertebral artery and neurological injuries. If the patient with AS had cervical kyphosis, skull traction should not be used for neck traction (25). However, skull traction could not completely reduce the AS cervical spine fractures in the absence of general anesthesia. Although skull traction has been believed to be safe under general anesthesia, Sornatosensoryevokedpotentials (SEPs) should be used to monitor neurologic signals (26).

In this research and our clinical work, our data proposed that the halo-vest could be the optimal solution for early therapy of cervical spine fracture in AS patients. In this study, 25 patients with AS cervical spine fracture were treated with the halo-vest, and all patients successfully reduced the fracture site. Short-term use of the halo-vest can instantly stabilize the patient's neurological state, relieve spinal cord compression, and improve the cervical spine sequence. The advantages of the halo-vest include more precise cervical spine positioning, success in maintaining reduction, effective immobilization, and the ability to allow early mobilization of the patient (27). Through halo-vest, patients can receive safer and more effective surgical treatment after cervical spine reduction. Because of the effective reduction before operation that cloud shorten the time of surgical reduction, patients with halo-vest reduction have less bleeding and shorter operation time than patients with skull traction reduction. Therefore, surgeons should pay more attention to the type of cervical fracture to comprehensively consider the selection of appropriate surgical methods.

Although the halo-vest offers the best stabilization than other external immobilizers, it does not rigidly immobilize the cervical spine. Lee et al. reported that the halo device caused spinal instability, and increased range of motion was not conducive to bone fusion and self-healing (28). The halo-vest increased the cervical spine range of motion at the C2/C3 segment in the general population by 42%. The lower the cervical spine segment, the greater the range of motion, accompanied by a poorer fixation effect (29). Not only that, but in elderly patients, external fixation also carries risks. Prolonged external fixation is associated with a high risk of detachment or reduction, skin ulceration, and lung problems (30, 31). Some studies reported that surgical treatment in the early stage of the disease has a lower incidence of pseudarthrosis and neurologic deficits and a better prognosis (15, 32). Therefore, to avoid complications related to conservative treatment, especially the needle site infection and the deterioration of the nervous system, the patient should still undergo surgical treatment as soon as possible to stabilize the condition. Therefore, the authors believe that the main purpose of preoperative halo-vests is temporary external stabilization of the injured cervical spine and that early surgical intervention should be performed regardless of the results of preoperative halo-vests. However, patients with AS may experience postural deterioration and iatrogenic spinal fractures during surgery. Halo-vest reduction should be used to avoid movement in patients with AS fractures and correct spinal flexion deformity after AS trauma. It is easier to remove bone fragments and complete decompression after cervical spine reduction with halo-vests (33, 34). This study found that the adjustment and control of the halo-vest stent can complete the three-dimensional adjustment of the cervical spine, improve the success rate of reduction, and effectively avoid secondary nerve damage.

The current study also has limitations. First, the sample size is relatively small, and the follow-up was relatively short in this study. Second, as with any retrospective study, selection and measurement can be biased. The retrospective results from a single-center should be prospectively verified by multicenter and randomized controlled studies. Therefore, future studies should be prospective, randomized, controlled, and longer.

## Conclusion

In summary, skull traction is advocated because of its ease of use, whereas a halo-vest can be preferred in AS patients with cervical spine fractures. Successful reduction and satisfactory neurological recovery can be achieved by halo-vest for cervical fracture dislocation in patients with AS. It provides safe, simple, and accurate cervical traction and cervical fixation for AS patients after fracture. After cervical spine reduction, patients can receive safer and more effective surgical treatment.

## Data Availability

The datasets presented in this study can be found in online repositories. The names of the repository/repositories and accession number(s) can be found in the article.
